# Feature Binding of Common Everyday Items Is Not Affected by Age

**DOI:** 10.3389/fnagi.2017.00122

**Published:** 2017-05-10

**Authors:** Serge Hoefeijzers, Alfredis González Hernández, Angela Magnolia Rios, Mario A. Parra

**Affiliations:** ^1^Department of Psychology, School of Social Sciences, Heriot-Watt UniversityEdinburgh, UK; ^2^DENEUROPSY, Surcolombiana UniversityHuila, Colombia; ^3^Department of Psychology, Human Cognitive Neuroscience and Centre for Cognitive Ageing and Cognitive Epidemiology, University of EdinburghEdinburgh, UK; ^4^Alzheimer Scotland Dementia Research Centre, University of EdinburghEdinburgh, UK; ^5^Scottish Dementia Clinical Research Network, University of EdinburghEdinburgh, UK; ^6^Facultad de Psicologia, Universidad Autónoma del CaribeBarranquilla, Colombia

**Keywords:** visual working memory binding, ageing, cross-cultural validity, cognition, neuropsychological assessment, Feature binding, Common everyday items

## Abstract

There is a surge of studies confirming that old age spares the ability to bind in visual working memory (VWM) multiple features within singular object representations. Furthermore, it has been suggested that such ability may also be independent of the cultural background of the assessed individual. However, this evidence has been gathered with tasks that use arbitrary bindings of unfamiliar features. Whether age spares memory binding functions when the memoranda are features of everyday life objects remains less well explored. The present study investigated the influence of age, memory delay, and education, on conjunctive binding functions responsible for representing everyday items in VWM. We asked 32 healthy young and 41 healthy older adults to perform a memory binding task. During the task, participants saw visual arrays of objects, colours, or coloured objects presented for 6 s. Immediately after they were asked either to select the objects or the colours that were presented during the study display from larger sets of objects or colours, or to recombine them by selecting from such sets the objects and their corresponding colours. This procedure was repeated immediately after but this time providing a 30 s unfiled delay. We manipulated familiarity by presenting congruent and incongruent object-colour pairings. The results showed that the ability to bind intrinsic features in VWM does not decline with age even when these features belong to everyday items and form novel or well-known associations. Such preserved memory binding abilities held across memory delays. The impact of feature congruency on item-recognition appears to be greater in older than in younger adults. This suggests that long-term memory (LTM) supports binding functions carried out in VWM for familiar everyday items and older adults still benefit from this LTM support. We have expanded the evidence supporting the lack of age effects on VWM binding functions to new feature and object domains (i.e., everyday items). We have confirmed that education does not negatively impact on such ability at old age. Such results have important implications for the selection of culturally unbiased tests to screen for abnormal ageing trajectories.

## Introduction

Associative memory functions, such as those needed to remember the location of objects, decline with age. This effect is thought to underlie age-related impairments in retaining associations in long-term memory (LTM) (Chalfonte and Johnson, 1996), which in turn affects older people's episodic memory (Naveh-Benjamin et al., 2004, 2007; Old and Naveh-Benjamin, 2008; Shing et al., 2010; Peterson and Naveh-Benjamin, 2016). However, age-related associative memory deficits have also been observed over intervals of seconds, suggesting that the ability to form and maintain associations in short-term memory (STM) also declines with age (Cowan et al., 2006; Mitchell et al., 2006; Chen and Naveh-Benjamin, 2012; Cowan, 2016; Peterson and Naveh-Benjamin, 2016).

Whereas age affects the retention of associations both in LTM and in STM (but see Bopp and Verhaeghen, 2009; Pertzov et al., 2015), it seems to spare the ability to hold surface features, such as shapes and colours, bound within integrated objects in STM (Brockmole et al., 2008; Parra et al., 2009b; Brown and Brockmole, 2010; Brockmole and Logie, 2013; Isella et al., 2015; Read et al., 2016; Brown et al., 2017). Rhodes and colleagues argued for a distinction between binding of extrinsic features (i.e., linking of distinct items or contextual features accompanying an item) and binding of features that define the intrinsic characteristics of an object (i.e., within-item binding) (see also Allen et al., 2013; Rhodes et al., 2015). Binding objects' intrinsic features appears to be an automatic process (Allen et al., 2006, 2009; Karlsen et al., 2010; but see Shen et al., 2015; Gao et al., 2017) that is largely spared by age (Parra et al., 2009b; Brown and Brockmole, 2010; Isella et al., 2015; Rhodes et al., 2015; Read et al., 2016; Brown et al., 2017). In contrast, binding extrinsic features requires more cognitive resources (e.g., associative functions of the medial temporal lobe), which appear to be more susceptible to the effects of age (Mitchell et al., 2006).

Although general visual STM (VSTM) abilities do decline with older age (Reuter-Lorenz and Sylvester, 2005), the ability to integrate multiple surface features into singular object representations remains preserved across the lifespan (Brockmole et al., 2008; Parra et al., 2009b,c; Brown and Brockmole, 2010; Brockmole and Logie, 2013; Isella et al., 2015; Rhodes et al., 2015; Read et al., 2016; Brown et al., 2017). To ascertain that binding is selectively compromised, one needs to demonstrate that memory for the constituent parts is preserved. A psychometrically valid memory binding paradigm should assess the cost of binding against the cost of processing single features (shape and colour). Such a cost has proved to remain stable across the lifespan.

However, the same VSTM binding functions that have proved insensitive to normal ageing have been found to be dramatically affected in patients with Alzheimer's disease (AD) both when they recall (Parra et al., 2009a) or recognise (Parra et al., 2010a) feature conjunctions. Patients with AD show a disproportional cost of binding relative to healthy controls. This impairment becomes apparent in otherwise asymptomatic carriers of the E280A presenilin-1 gene mutation who will inevitably develop familial AD more than 10 years prior to the onset of dementia (Parra et al., 2010b, 2011). This indicates that VSTM binding holds marker properties to identify the pre-clinical stages of AD. It is worth noting that binding intrinsic features in VSTM is not affected by depression (Parra et al., 2010a) or other non-AD dementias (i.e., FTD, PD, VasD, DLB) (Della Sala et al., 2012), making it both sensitive and specific for AD. For a cognitive test to be considered a reliable marker for AD, it should also be insensitive to the cultural background of the assessed individual (Logie et al., 2015). Parra et al. (2011) suggested that feature binding in VSTM also appears to meet this criterion. The authors compared data from a change detection task of patients with sporadic and familial AD in samples recruited in Scotland and in Colombia, as well as data from healthy controls also recruited in both countries. Mean performance of patients and controls across countries did not differ significantly despite significant differences of age and education between samples across countries. In that earlier study, as well as in most of the above-mentioned studies investigating VSTM binding, meaningless combinations of random shapes and colours were the memoranda. It remains unknown whether age and education impair feature binding of common everyday items.

The present study investigated whether the ability to process in memory congruent and incongruent combinations of common surface features over short and long retention intervals is differentially affected in older adults with a low educational background. The results from studies by Parra and colleagues in healthy ageing (Brockmole et al., 2008; Parra et al., 2009b) and those in AD (Parra et al., 2010a,b) involved tasks assessing memory for coloured-shapes which are unfamiliar making them difficult to name or rehearse. Whether binding functions carried out in visual working memory (VWM) which support the integration of common features into familiar everyday items are also insensitive to age and education is yet unknown. Moreover, whether such factors (i.e., age and education) spare the representations of complex items in memory over longer memory delays is another question that needs investigation. Recent evidence from Chen and Naveh-Benjamin (2012) shows that age-related associative memory deficits for extrinsic features (i.e., face-scene association) are evident over both short (seconds) and longer delay intervals (minutes). As we used familiar everyday items, the support that VWM binding functions would receive from LTM would be greater in the context of the present study than in that of earlier studies (Brockmole et al., 2008; Parra et al., 2009b). Such a support is expected to render the influence of factors, such as age and education even more informative. We further manipulated the availability of such a support by presenting congruent (e.g., typical red apple) and incongruent object-colour pairings. We used a paradigm which assesses reconstruction rather than simple recognition (Parra et al., 2009c). Previous studies using similar versions of this paradigm have reported a lack of age effects on VSTM binding (Brockmole and Logie, 2013; van Geldorp et al., 2015). Reconstruction is a more challenging task than recognition as it involves aspects of both recall and recognition of previous experiences (van Geldorp et al., 2015). Such a paradigm would increase the likelihood of identifying effects of age on VWM binding functions under the different experimental conditions investigated here.

Based on core features of the memory binding paradigm used in this study we made the following predictions. Reconstruction accuracy would be significantly better for congruent object-colour bindings than for incongruent bindings regardless of age, as VWM binding functions would receive more support from LTM. In line with previous studies, no age-related binding deficits would be observed for familiar everyday items. Longer delay periods would have lesser impact on younger than on older adults. This is because the use of familiar items in our task may facilitate access to LTM and rehearsal over longer delays, functions known to be sensitive to normal ageing (Poon and Fozard, 1980; Nielsen-Bohlman and Knight, 1995; Brown et al., 2012).

## Materials and methods

We investigated the ability of healthy young adults and healthy older adults to hold congruent (i.e., white hen) and incongruent (i.e., purple bread) combinations of common surface features and to recognise them immediately after encoding (i.e., 0 ms delay) and after a long retention interval (i.e., 30 s delay). To this aim, we relied on a mixed design (i.e., ANOVA) with age as the between-subjects factor and congruency and delay as the repeated measures, each with two levels. Moreover, due to the heterogeneity of this population with regard to their educational background, we capitalised on this opportunity to explore whether and to what extent such a demographic factor affected performance across conditions and age groups. This was achieved via ANCOVA.

### Participants

A group of 32 healthy young adults (25 female) and a group of 41 healthy older adults (27 female) were recruited for the study. The study took place at the Psychology Department of the Surcolombiana University, Colombia. Younger and older participants were recruited from the university setting and from the community. They were invited via local media advertisements or were approached directly by members of the research group. All the participants were fully informed about the study. Informed consent was obtained from each participant according to the Declaration of Helsinki (World Medical Association, 2001). The study was reviewed and approved by the University's Ethics Committee.

Younger and older adults' groups significantly differed in age and education. Healthy older adults had significantly fewer years of education than younger adults. The healthy condition of our sample was ascertained via a brief neuropsychological assessment (see Table 1). In fact, the sample of healthy older adults collected for this study outperformed the local norms (Aguirre-Acevedo et al., 2007).

**Table 1:** **Demographic and neuropsychological data and results from the statistical comparisons**.

**Variable**	Healthy young adults (*n* = 32)/Or Norms (^*^) Mean (SD)	**Healthy elderly (*n* = 41) Mean (SD)**	***t* (p); Effect size (*r*); Power (β)**
Age (years)	33.44 (8.36)	70.22 (7.34)	−19.99 (<0.001); 0.92; 1.00
Education (years)	12.03 (5.26)	4.85 (3.21)	6.80 (<0.001); 0.70; 1.00
MMSE^*^	27.76 (1.77)	27.70 (2.10)	0.16 (0.877); 0.01
World List Learning (Total Recall)^*^	3.55 (1.99)	6.03 (2.20)	6.21 (<0.001); 0.43
World List Learning Recognition^*^	7.70 (2.31)	9.8 (0.48)	23.73 (<0.001); 0.52
ROF-Copy^*^	18.08 (6.16)	27.12 (8.56)	5.78 (<0.001); 0.43
ROF-Recall^*^	6.51 (3.88)	9.77 (7.42)	2.40 (<0.023); 0.20

* *Values taken from the norms (Aguirre-Acevedo et al., 2007), these were compared with the sample's values using one-sample t-tests*.

### The VSTM task

**Figure 1:**
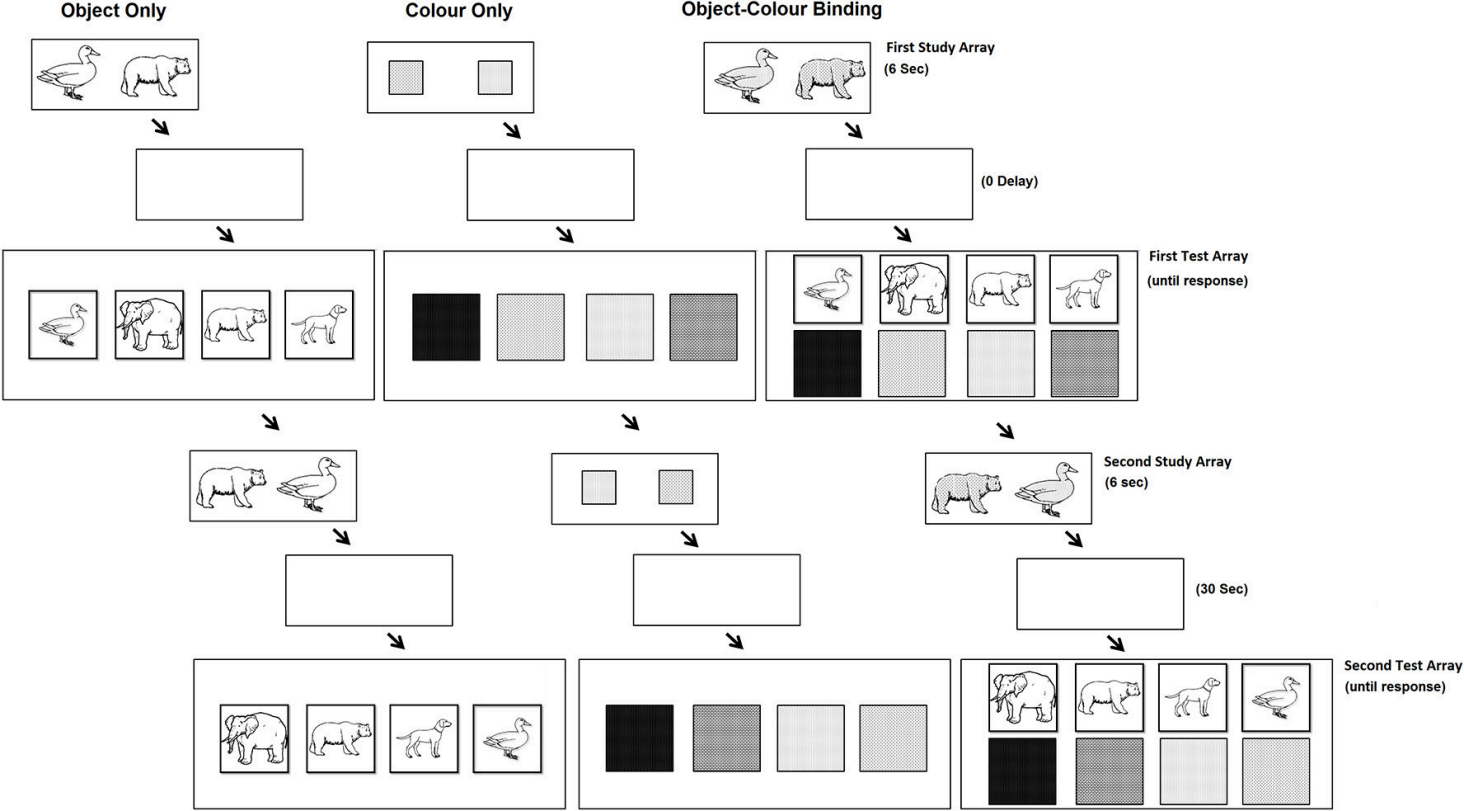
**The experimental conditions and trial sequences used in our experiment (see text for description)**.

The paradigm applied in this study was based on paradigms used by (Parra et al., 2009c). Two sets of 10 nameable colours (Red, Blue, Green, Brown, Orange, Yellow, Purple, Silver, Turquoise, and Pink) and 20 nameable objects were used. The set of objects consisted of 10 man-made objects (saw, bread, cross, shoe, glove, hammer, hat, lamp, light bulb, frying pan) and 10 living objects (duck, crocodile, arm, baby, bear, bee, cat, cow, dog, hen). Objects were taken from the International Picture Naming Project (http://crl.ucsd.edu/~aszekely/ipnp/). The stimuli were presented on a 15″ computer screen. As shown in Figure 1, the task consisted of three test conditions: the Object Only condition, the Colour Only condition and the Object-Colour Binding condition. For each test condition, participants were presented with a first study array which was followed immediately after by the first test array (immediate recognition). After participants' response, the same study array was presented again this time followed by a 30 s unfilled retention interval before the second test array was presented.

#### First study array

In the study array, participants were presented with objects, colours or coloured objects. To control for memory load, as determined by the number of to-be-remembered features, we kept the number of features constant for each test condition and varied the number of objects. Participants were presented with study arrays consisting of 4 objects (Object Only condition), 4 colours (Colour Only condition), or 2 combinations of objects and colours (Object-Colour Binding condition). The study array was presented for 1.5 s per feature (6 s in total). Participants were instructed to pay close attention to the presented items and to try to memorise them.

#### Test array (immediate)

Immediately after the study array a test array was presented. In the condition assessing memory for single features (i.e., the Object Only condition and the Colour Only condition) the test array presented twice as many items as the study array. Half of these items corresponded to those previously seen while the other half were items not presented in the study array. Participants were requested to select, using the mouse, the objects or the colours they had seen in the study array. In the condition assessing memory for Object-Colour Binding, the test display presented two separate arrays of items. One array consisted of the same objects previously seen plus the same number of new distracter objects, and the second array consisted of the same colours previously seen plus the same number of new distracter colours. Participants were requested to select, using the mouse, the objects they had seen in the study array with their corresponding colours (i.e., choose each object and its colour). For the object-colour binding condition we designed two separate blocks of trials. One block presented congruent combinations of objects and colours (e.g., yellow hen, brown bear, black shoes, silver frying pan) and the other presented incongruent combinations (e.g., pink bee, red crocodile, green hammer, purple saw). Stimuli congruency ratings was high for both groups (see post VSTM Task Congruency Test below).

#### Second study array

After immediate recognition, for which feedback was not provided, the same study display was presented again as described above. This was followed by a 30 s unfilled retention interval. Presenting the same study array a second time assured that the to-be-remembered items were well presented in VWM prior to the 30 s delay interval as they could have been overwritten by items shown in the First Test array. As bindings held in VWM are fragile (quickly overwritten), we can assume that the information held during this longer retention interval was likely sourced from the Second Study array rather than from cumulative knowledge originating through the First and Second Study arrays (see Colzato et al., 2006; Logie et al., 2009).

#### Second test array (30 s memory delay)

After this longer delay, participants were presented with the test display again. In the Second Study array and delayed Test array, items were presented in new random locations. This manipulation was introduced to prevent the use of location as a memory cue.

Each test condition consisted of 8 trials (i.e., 4 blocks of 8 trials each). For the Objects only condition and the Object-colour binding condition, 4 of the 8 trials consisted of man-made objects. The remaining 4 trials consisted of living objects. Trials were randomised and experimental conditions were delivered in a counterbalanced order across participants. It is worth noting that the presentation time of the encoding display and delay periods (0 s and 30 s) used in our paradigm are not features of a typical working memory task. Long encoding times (1.5 s per feature) were used to enable an accurate representation of the to-be-remembered information. Such a procedure has been extensively used in previous studies (Parra et al., 2009c, 2015). Active recognition is not susceptible to the influence of iconic memory as change detection tasks may be. Moreover, retrieval functions during active recognition take longer to operate than during “same/different” change detection tasks, making immediate retention scores (0 ms delay) accurate measures of VWM (see Brockmole and Logie, 2013). Lastly, we included a 30 s delay interval which we left unfilled to create opportunity for rehearsal. As this function declines with age (Brown et al., 2012), we were interested in investigating whether such a reliance would reveal dissociations of VWM for features and bindings of familiar everyday objects.

### Post VSTM task congruency test

After the VWM task, participants were presented with a screening test which assessed their perceived congruency of the coloured objects. To this aim each participant was simultaneously presented with two coloured objects. The objects and the colours had all been previously used in the VWM Task. The object presented in each screen was the same but it was shown with two different colours. For all the trials the object was paired with a congruent and an incongruent colour (side to side counterbalancing their position). In half of the trials one of the two combinations was that defined as congruent in the VWM task. In the other half one of the two combinations was that defined as incongruent in the VWM task. The participants were asked to select, using the mouse, the object of the pair that they thought was presented in its typical colour. We calculated the percentage of congruency. This task enabled us to ascertain that the intended congruency of the to-be-remembered bindings was indeed the perceived congruency. Independent-sample *t*-tests confirmed that this was the case. Both young and older adults obtained high congruency scores for the congruent and incongruent object-colour bindings that were used in the VWM task (90.63%, SD = 8.40%; 86.72%, SD = 12.91%, respectively) which did not differ statistically [*t*_(70)_ = 1.479, *p* = 0.144, *r* = 0.17, β = 0.34].

### Scoring procedures

Percentage Correct responses. The participant's percentage of correct responses was calculated for each test condition for both the immediate and 30 s delay test. Percentage Correct responses was calculated via the following equation: (# of objects, colour or object-colour combination – correctly recognised/total studied items) × 100. For the Object-Colour Binding condition, items were scored (i.e., a correct response) if the object and its corresponding colour were correctly selected. For this condition we generated two scores, one for congruent bindings and one for incongruent bindings.

While participants' judgment of congruent items was higher for living items than for man-made items, there was neither an effect of Group nor a Group × Category interaction (see Supplementary Table 1). Moreover, further exploratory analysis revealed that response accuracy (i.e., the Percentage correct responses) for congruent and incongruent items did not differ significantly across living or man-made items (see Supplementary Figure 1). We therefore collapsed the data across living and man-made categories and focused on the congruency effect only.

### Statistical analyses

Independent-sample or one-sample *t*-tests were conducted to compare demographic and neuropsychological data across groups (see Table 1). We applied a mixed ANOVA to examine the Percentage Correct responses across the within-subjects factors Test Condition (Objects Only vs. Colour Only vs. Congruent Object-Colour Binding vs. Incongruent Object-Colour Binding) and Delay (Immediate vs. 30 s Delay), and the between-subjects factor Group (Younger vs. Older). When the interactions were found to be significant, *post-hoc* contrasts were carried out across groups for each test condition separately (i.e., 4 comparisons) and across conditions for each group separately (6 comparisons per group). This was done for both the immediate and 30 s delay test (see Table 2). To avoid type-I error we used Bonferroni correction (alpha level: 0.05/32 = 0.001). We also implemented a mixed ANOVA collapsing performance across Delay (within-subjects factor: Test Condition; between-subjects factor: Group). Again, when the interactions were found to be significant, *post-hoc* contrasts were carried out across groups for each test condition separately (i.e., 4 comparisons) and across conditions for each group separately (6 comparisons per group). Bonferroni correction (alpha level: 0.05/16 = 0.003) were used to avoid type-I error. To further investigate the effect of congruency on memory binding, additional mixed ANOVAs (within-subjects factor: Test Condition; between-subjects factor: Group) were conducted with one of the 2 binding conditions (Incongruent Object-Colour Binding or Congruent Object-Colour binding) excluded from the analysis (see Supplementary Table 2). Moreover, an additional ANCOVA was carried out by adding “Education” as a covariate to the above described models. For the four *post-hoc* comparisons investigating the Percentage Correct responses between groups for each test condition, we adjusted the alpha level to 0.0125. The effect of Education on binding congruency was further examined by carrying out both a mixed ANOVA and an ANCOVA controlling for Education using Group (Younger vs. Older) as between-subjects factor and Test Condition (Congruent Object-Colour Binding vs. Incongruent Object-Colour Binding) as within-subjects factor (see Supplementary Table 3). Effect sizes for the ANOVAs and ANCOVAs were determined using partial eta-squared $\eta_{\text{p}}^{2}$, where 0.14 is a large effect (Stevens, 2002). For *t*-tests we used *r*, where 0.37 reflects a large effect size (Cohen, 1988). The alpha level was set to 0.05 for all analyses (except for the *post-hoc* comparisons), which were conducted in IBM SPSS Statistics 22.

**Table 2:** **Results from paired-sample ***t***-tests contrasting performance between test conditions across healthy young and older adults for the immediate and 30 s test delay**.

**PERFORMANCE ACROSS GROUPS FOR EACH TEST CONDITION AT THE IMMEDIATE AND 30 S TEST DELAY**				
**Test condition**	**Immediate delay**		**30 s delay**	
	*t* (p); Effect size (*r*); Power (β)		*t* (p); Effect size (*r*); Power (β)	
Colours	**6.74 (<0.001); 0.66; 1.00**		**6.05 (<0.001); 0.61; 1.00**	
Objects	**6.61 (<0.001); 0.62; 1.00**		**5.65 (<0.001); 0.57; 1.00**	
Binding Congruent Objects	2.21 (0.030); 0.26; 0.64		**3.59 (0.001); 0.41; 0.95**	
Binding Incongruent Objects	**4.11 (<0.001); 0.47; 0.98**		**6.30 (<0.001); 0.65; 1.00**	
**PERFORMANCE ACROSS TEST CONDITION FOR EACH GROUP AT THE IMMEDIATE AND 30 S TEST DELAY**				
	**Immediate delay**		**30 s delay**	
**Test condition**	**Healthy young adults**	**Healthy older adults**	**Healthy young adults**	**Healthy elderly**
	*t* (p); Effect size (*r*); Power (β)	*t* (p); Effect size (*r*); Power (β)	*t* (p); Effect size (*r*); Power (β)	*t* (p); Effect size (*r*); Power (β)
Colours vs. Objects	−0.78 (0.443); 0.14; 0.11	−2.27 (0.029); 0.34; 0.61	−1.07 (0.295); 0.19; 0.17	−1.29 (0.205); 0.20; 0.25
Colours vs. Binding Congruent objects	−1.06 (0.299); 0.19; 0.17	−**5.28 (<0.001); 0.65; 1.00**	−3.04 (0.005); 0.48; 0.82	−**5.59 (<0.001); 0.67; 1.00**
Colours vs. Binding Incongruent objects	−0.77 (0.447); 0.14; 0.11	−1.32 (0.195); 0.21; 0.25	−**4.80 (<0.001); 0.65; 1.00**	−2.86 (0.007); 0.42; 0.81
Objects vs. Binding Congruent objects	−0.27 (0.793); 0.05; 0.04	−**3.98 (<0.001); 0.54; 0.98**	−2.15 (0.040); 0.36; 0.53	−**3.88 (<0.001); 0.53; 0.97**
Objects vs. Binding Incongruent objects	0.05 (0.958); 0.01; 0.03	−0.03 (0.980); 0.00; 0.03	−**4.02 (<0.001); 0.59; 0.97**	−1.39 (0.174); 0.22; 0.28
Binding Congruent objects vs. Binding Incongruent objects	0.34 (0.738); 0.06; 0.05	**3.67 (0.001); 0.51; 0.95**	−0.97 (0.340); 0.17; 0.15	**3.54 (0.001); 0.49; 0.94**

*The alpha level was set to 0.05 for all mixed-factor ANOVAs. In order to avoid type-I error, we adjusted the alpha level to 0.001, using Bonferroni correction, i.e., 0.05/32 comparisons (For each delay interval: 6 within-group comparisons per test group, i.e., each comparison is between 2 of the 4 test conditions; 4 between group comparisons for each test condition–see statistical analysis). The values in bold represent statistical significant findings*.

## Results

Table 1 shows the age and years of education of the two groups. Healthy older adults had significantly fewer years of education than the younger adults [*t*_(48.52)_ = 6.80, *p* < 0.001, *r* = 0.70, β = 1.00].

### Short-term memory tasks

**Figure 2:**
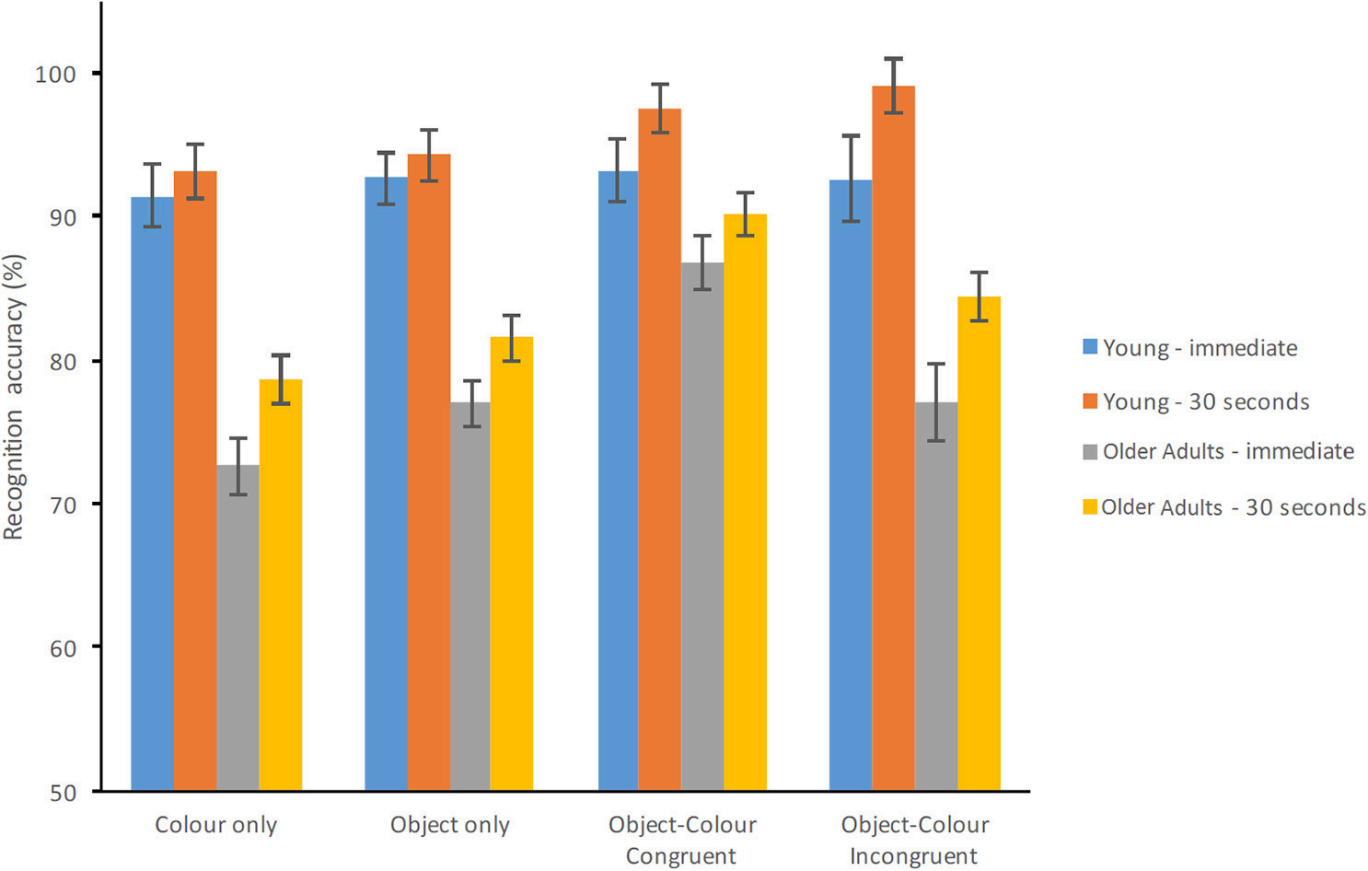
**Percentage of correct recognition for healthy young and older adults for each test condition and test delay (Error bars represent the standard error of the mean)**.

The ANOVA model (Group × Test Condition × Delay) revealed a significant effect of Group [*F*_(1, 70)_ = 46.559, *p* < 0.001, $\eta_{\text{p}}^{2}$ = 0.399, β = 1.00] whereby healthy older adults performed significantly poorer than young adults (see Figure 2). Test Condition also resulted in a significant main effect [*F*_(3, 210)_ = 15.368, *p* < 0.001, $\eta_{\text{p}}^{2}$ = 0.180, β = 1.00] whereby the percentage of correct responses was the highest for congruent Object-Colour Binding (91.88%, SE = 1.11%) followed by incongruent Object-Colour Binding (88.25%, SE = 1.46%), and Object Only (86.36%, SE = 1.05%). The percentage of correct responses for the Colour Only condition was the lowest (83.96%, SE = 1.22%). There was a main effect of Delay [*F*_(1, 70)_ = 29.042, *p* < 0.001, $\eta_{\text{p}}^{2}$ = 0.293, β = 1.00] whereby response accuracy improved significantly after the 30 s delay compared to immediate recognition. The interaction between Group and Test condition was significant [*F*_(3, 210)_ = 6.483, *p* < 0.001, $\eta_{\text{p}}^{2}$ = 0.085, β = 0.969]. Neither the Group × Delay [*F*_(1, 70)_ = 1.224, *p* = 0.272, $\eta_{\text{p}}^{2}$ = 0.017, β = 0.194] nor the Group × Test Condition × Delay interaction [*F*_(3, 210)_ = 0.766, *p* = 0.514, $\eta_{\text{p}}^{2}$ = 0.011, β = 0.213] were found to be significant.

*Post-hoc* comparisons carried out across Group for each Condition and Delay are shown in Table 2. Young adults outperformed the older adults on all test conditions, except for congruent Colour-Object Binding with 0 s delay. Table 2 also shows performance across Condition for each Group at each Delay. No significant differences between test conditions were observed for young adults at 0 s delay. However, young adults performed significantly better on the Incongruent Colour-Object Binding condition compared to the Colour only or Object Only condition with 30 s delay. Healthy older adults performed significantly better on the Congruent Colour-Object binding condition compared to the other 3 test conditions. This was the case regardless of delay. As Delay did not interact with Group nor did it modify the key Group × Test Condition interaction, we collapsed the data across this factor and ran a two way ANOVA with Group and Test Condition.

**Figure 3:**
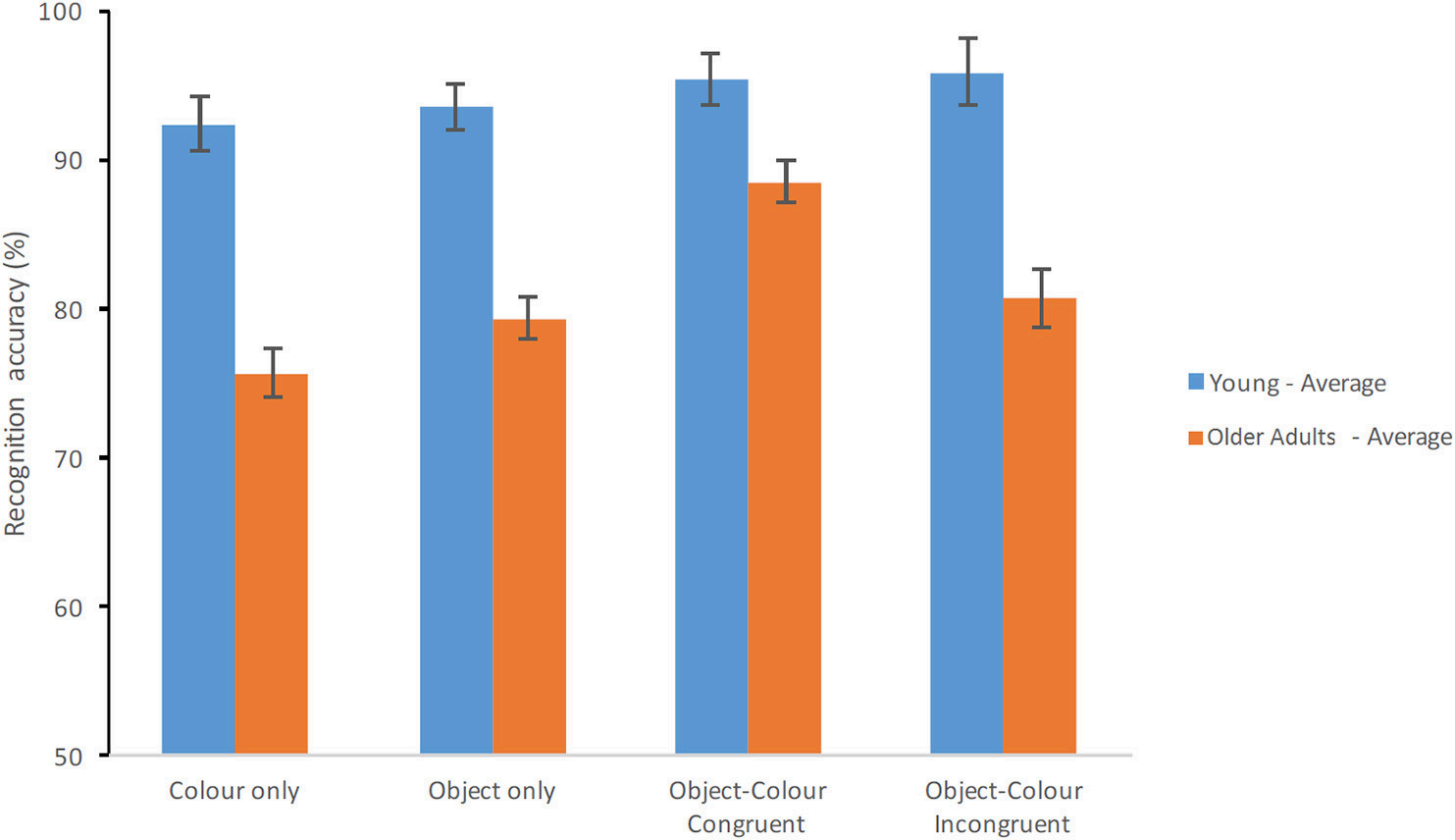
**Percentage of correct recognition averaged across test delays**. Average scores of young adults and healthy older adults are shown for each test condition (Error bars represent the standard error of the mean).

Figure 3 shows the average Percentage Correct responses of healthy young and older adults across the four conditions of the STM Task. There was a main effect of Group [*F*_(1, 70)_ = 46.559, *p* < 0.001, $\eta_{\text{p}}^{2}$ = 0.399, β = 1.00] whereby healthy older adults performed significantly poorer than younger adults. The main effect of Condition was also significant [*F*_(3, 210)_ = 15.368, *p* < 0.001, $\eta_{\text{p}}^{2}$ = 0.18, β = 1.00] whereby congruent Object-Colour Binding > incongruent Object-Colour Binding > Objects Only > Colour only (see previous analysis for recognition scores). Crucially, the Group × Test Condition interaction was significant [*F*_(3, 210)_ = 6.483, *p* < 0.001, $\eta_{\text{p}}^{2}$ = 0.085, β = 0.969].

*Post-hoc* comparisons carried out across Group for each Test Condition separately showed that young adults outperformed the older adults on all tests (see Table 3). Performance across Test Condition for each Group (6 comparisons per group) showed that for healthy young adults performance did only differ significantly between the Colour Only condition and the Object–Colour Binding condition for incongruent bindings, with the percentage of correct responses for incongruent bindings being better than for colours only (see Figure 3). In contrast, for healthy older adults, performance on the Object-Colour binding condition for congruent object-colour bindings was significantly better than on all the other test conditions. No other contrasts revealed significant differences (see Table 3). In sum, these results suggest that the interaction was driven by a significantly better performance of older adults on the Object-Colour binding condition congruent trials relative to the other conditions, an effect that was not apparent in younger adults. This assumption is further supported by the findings of an additional 2 (age group) × 3 (test condition) ANOVAs shown in Supplementary Table 2. Here we included either the Congruent Object-Colour binding condition or the Incongruent Object-Colour binding condition as “binding” variable in the ANOVA (i.e., the included test conditions were: Objects only, Colour-only and either Incongruent Object-Colour binding or Congruent Object-Colour binding). The Group × Condition interaction was observed if the Congruent Object-Colour binding condition [*F*_(2, 140)_ = 9.082, *p* < 0.001, $\eta_{\text{p}}^{2}$ = 0.115, β = 0.973] was included in the analysis, but not when the Incongruent Object-Colour binding was included [*F*_(2, 140)_ = 0.517, *p* = 0.598, $\eta_{\text{p}}^{2}$ = 0.007, β = 0.134]. The significant interaction was driven by increased accuracy of older adults in the congruent binding condition.

**Table 3:** **Results from paired-sample ***t***-tests contrasting performance between test conditions across healthy young and older adults**.

**Performance across groups for each test condition**		
**Test condition**	***t* (p); Effect size (*r*); Power**	
Colours	**7.29 (<0.001); 0.69; 1.00**	
Objects	**7.07 (<0.001); 0.65; 1.00**	
Binding Congruent Objects	**3.30 (0.002); 0.39; 0.91**	
Binding Incontruent Objects	**5.63 (<0.001); 0.61; 1.00**	
**Performance across test condition for each group Test condition**	**Healthy young adults**	**Healthy older adults**
	*t* (p); Effect size (*r*); Power (β)	*t* (p); Effect size (*r*); Power (β)
Colours vs. Objects	−1.11 (0.275); 0.20; 0.19	−2.02 (0.05); 0.34; 0.51
Colours vs. Binding Congruent objects	−2.39 (0.023); 0.39; 0.65	−**6.67 (<0.001); 0.77; 1.00**
Colours vs. Binding Incongruent objects	−**3.88 (0.001); 0.57; 0.97**	−2.41 (0.021); 0.40; 0.66
Objects vs. Binding Congruent objects	−1.40 (0.172); 0.24; 0.28	−**4.66 (<0.001); 0.64; 1.00**
Objects vs. Binding Incongruent objects	−2.14 (0.040); 0.36; 0.56	−0.67 (0.508); 0.12; 0.10
Binding Congruent objects vs. Binding Incongruent objects	−0.41 (0.683); 0.07; 0.06	**4.56 (<0.001); 0.63; 0.99**

*Remark: The alpha level was set to 0.05 for all mixed-factor ANOVAs. In order to avoid type-I error, we adjusted the alpha level to 0.003, using Bonferroni correction, i.e., 0.05/16 comparisons (6 within-group comparisons per test group, i.e., each comparison is between 2 of the 4 test conditions; 4 between group comparisons for each test condition – see statistical analysis). The values in bold represent statistical significant findings*.

**Figure 4:**
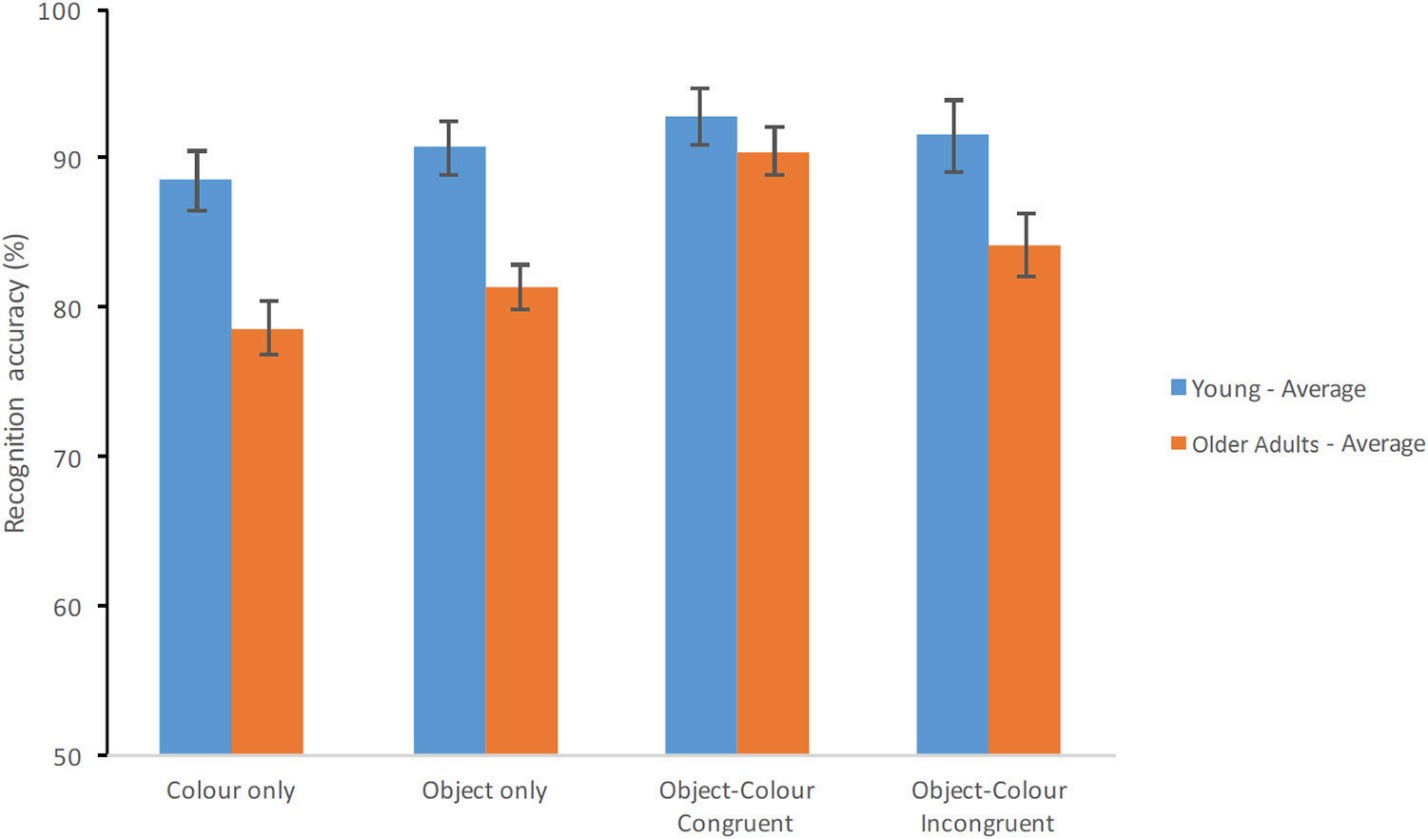
**Percentage of correct recognition averaged across test delays when the analysis was controlled for education**. Average scores of young adults and healthy older adults are shown for each test condition (Error bars represent the standard error of the mean).

Finally, the ANCOVA model controlling for the effects of education showed that this covariate had a significant effect [*F*_(1, 69)_ = 16.134, *p* < 0.001, $\eta_{\text{p}}^{2}$ = 0.190, β = 0.977]. Nevertheless, education accounted neither for the effect of Group [*F*_(1, 69)_ = 9.821, *p* = 0.003, $\eta_{\text{p}}^{2}$ = 0.125, β = 0.871] nor Test Condition [*F*_(3, 207)_ = 5.3, *p* = 0.002, $\eta_{\text{p}}^{2}$ = 0.071, β = 0.928]. After controlling for education congruent Object-Colour Binding still yielded better performance (91.62%, SE = 1.07%) than incongruent Object-Colour Binding (87.82%, SE = 1.37%), than Objects Only (86.09%, SE = 1.00%) and Colour Only (83.58%, SE = 1.14%). Education did not interact with Test Condition [*F*_(3, 207)_ = 1.071, *p* = 0.362, $\eta_{\text{p}}^{2}$ = 0.015, β = 0.287]. However, controlling for Education did remove the Group × Condition interaction observed with the uncontrolled model [*F*_(3, 207)_ = 2.389, *p* = 0.07, $\eta_{\text{p}}^{2}$ = 0.033, β = 0.592]. As Figure 4 shows, overall performance of the younger group dropped after this manipulation which reduced group differences particularly on the Object-Colour Binding conditions. Of note, older adults' performance remained intact (see Figure 4). In fact, further contrasts across groups for each condition separately controlling for the effects of Education and type-I error showed that young adults still scored significantly better than healthy elderly on the Colour Only condition [*F*_(1, 69)_ = 11.110, *p* = 0.001, $\eta_{\text{p}}^{2}$ = 0.139, β = 0.908] and Objects Only condition [*F*_(1, 69)_ = 12.710, *p* = 0.001, $\eta_{\text{p}}^{2}$ = 0.156; β = 0.940] but not on conditions requiring memory binding [Colour-Object Binding for incongruent items: *F*_(1, 69)_ = 4.260, *p* = 0.043, $\eta_{\text{p}}^{2}$ = 0.058, β = 530; Colour-object binding for congruent items: *F*_(1, 69)_ = 0.671, *p* = 0.415, $\eta_{\text{p}}^{2}$ = 0.010, β = 0.127]. It is worth noting that controlling for Education significantly reduced the power of this analysis. Hence, the lack of interaction under this controlled analysis needs to be interpreted with caution. Nevertheless, it is worth noting that the variance controlled for by this analysis, originates, practically in its entirety, from the younger group as older adults' performance was virtually unaffected (if anything it improved).

The effects of education on age and binding congruency were further examined using a 2 (age group) × 2 (binding congruency: Incongruent vs. Congruent Object-Colour Binding) ANOVA and ANCOVA (see Supplementary Table 3). Interestingly, neither Age [*F*_(1, 69)_ = 2.808, *p* = 0.098, $\eta_{\text{p}}^{2}$ = 0.039, β = 0.379] nor the Age × Binding Congruency interaction yielded significant effects [*F*_(1, 69)_ = 3.258, *p* = 0.075, $\eta_{\text{p}}^{2}$ = 0.045, β = 0.429] once the analysis was controlled for Education. There was a significant main effect of Binding Congruency [*F*_(1, 69)_ = 9.347, *p* = 0.003, $\eta_{\text{p}}^{2}$ = 0.119, β = 0.854] with percentage of correct responses higher for the Congruent Object-Colour condition compared to the Incongruent Object-Colour condition (as shown in Figure 4). In other words, differences in Education across groups masked a potential benefit for congruent binding memory in young adults (which were highly educated). However, none of these effects suggested that older adults' VSTM binding abilities were poorer than those of younger adults.

In sum, the above data suggest that the ability to bind information in VSTM remains preserved in older adults with a low educational background. Older adults seem to capitalize on the support available from LTM when they perform online tasks (i.e., Congruent Bindings >>> Incongruent Bindings) and in the absent of such a support, they can still form and hold bindings in VWM at a cost no greater than that seen in younger adults.

## Discussion

The present study was set out to investigate whether the ability to hold in VSTM congruent and incongruent combinations of features of everyday objects over short and long retention intervals is differentially affected in older adults who have a low educational background. The key findings are: (1) the ability to bind intrinsic features in VWM does not decline with age even when these features belong to everyday items and form novel or well-known associations; (2) the impact of feature congruency on item-recognition appears to be greater in older than in younger adults; (3) preserved VWM binding abilities hold across delays; and (4) education aided performance in younger adults regardless of the memoranda an effect that was not apparent in older adult. We now discuss these findings in turn.

### Influences of LTM on VSTM binding across age

As suggested earlier, we found an overall impact of age on VWM but not differentially on binding functions carried out within this memory system (Brockmole et al., 2008; Parra et al., 2009b; Rhodes et al., 2015). Earlier studies on ageing and VWM binding used meaningless features or bindings which are unlikely represented in LTM or learn across trials (Colzato et al., 2006; Logie et al., 2009). The question of whether age also spares VWM binding functions than can be supported by LTM had not been explored previously. Moreover, unlike previous studies, in the current study we assessed VWM in conditions where load, as determined by the number of features, was kept constant across conditions. Olson and Jiang (2002) acknowledged that this setting may be appropriate to test the cost of binding. The authors suggested that the hypothesis of an object-based VWM (i.e., features are integrated with object representations at no extra cost) can be upheld if performance on binding conditions is better than that on single feature conditions. This was the case in our study, particularly in our older group.

For younger adults, the percentage of correct responses did not differ significantly between congruent and incongruent colour-object binding conditions. However, these findings should be interpreted cautiously as performance in this group was very high due to possibly a low memory load and the familiarity of the to-be-remembered items. Future work should follow this up where younger adults' and older adults' memory capacity is taken into account and the overall task difficulty is increased. Nevertheless, this finding suggests that holding novel bindings in VWM when memory load is kept within capacity may not add an additional cost. For older adults however, colour-object congruency did play a role. Indeed, the percentage of correct responses improved significantly for congruent combinations of objects and colours compared to when the object-colour combinations were incongruent (see Yang et al., 2013). Thus, our data suggest that LTM does support binding functions carried out in VWM and that such a support would come in handy in old age (e.g., Ruchkin et al., 2003; Postle, 2007). This preserved ability appears to be restricted to VWM as holding bindings of familiar features in LTM seems to be affected by age (Chalfonte and Johnson, 1996). Future studies should investigate the conditions leading to a shortage of this support in old age.

An alternative explanation for the effect of congruency observed in the older group could be that this age-related effect may result from interference (Sapkota et al., 2015). That is, LTM representations of everyday objects would impact either on congruent or incongruent object-colour parings. In the latter condition such existing knowledge may interfere during the reconstruction stage as similar objects had been previously experienced in congruent conjunctions within and outside the task context. As we age, we might be more susceptible to this form of interference as such an effect was not observed in the younger group. However, such an age-related interference seems unlikely as the Group × Condition interaction disappeared when the Congruent Object-Colour binding condition was excluded from the model (see Supplementary Table 2). In contrast, the Group by Condition interaction remained after excluding the Incongruent Object-Colour binding condition from the analysis. Thus, in older adults, LTM does not seem to interfere with incongruent memory binding, but rather support VWM binding of congruent object-colour parings.

Whether or not this benefit is exclusive for older adults remains debatable. In fact, our data suggest that the congruency effect on VWM binding seen in older adults might result, at least in part, from the influence of education (see Figure 4). Indeed, once the data was controlled for education, both groups seemed to benefit similarly from congruent Object-Colour parings (see Supplementary Table 3). Therefore, congruent memory binding might be beneficial for people with lower education in general. As we pointed out in the Results, controlling for Education reduced the power of the analysis and this may have led to a non-significant interaction. Future studies including larger sample sizes or groups with different educational levels will confirm this finding. Crucially, none of these effects indicated that age differently affects VWM binding abilities even when older adults have received fewer years of education than younger adults.

What do these findings tell us about ageing effects and current models of VWM memory (Baddeley, 2000)? When opportunities are created for a greater reliance on the episodic buffer by bridging the content of working memory with that of LTM and such a reliance supports the integration of surface features (i.e., the congruent Object-Colour binding condition), no age-related impairments on binding were observed. One might argue that such representations were more actively kept in a general working memory storage (e.g., the episodic buffer) responsible for linking the content of VWM to that of LTM (Baddeley, 2000). This evidence together with that from previous studies using canonical features of meaningless value, which are thought to be integrated in the visual spatial sketchpath (Baddeley et al., 2011), suggests that integrating features which require the support of the episodic buffer is also spared in ageing (at least for this type of representation).

### Influences of delay on VSTM binding across age

Interestingly, the percentage of correct responses improved significantly when the test array was presented 30 s after the second study array compared to when the test array occurred immediately after the initial study array; an effect that was evenly distributed across all test conditions and was independent of age. Age is known to affect memory over longer delays more dramatically than over shorter delays (Poon and Fozard, 1980; Nielsen-Bohlman and Knight, 1995). Older adults' rehearsal abilities are less efficient than that of younger adults (Brown et al., 2012). Taken together these earlier findings one would have predicted poorer performance of older adults over longer delays. It therefore remains questionable whether verbalisation (i.e., rehearsal) significantly influenced performance during the 30 s delay on either group. Indeed, older adults usually do not apply verbal strategies on VWM binding tasks unless they are encouraged to do so (Parra et al., 2009c). It could be that the high familiarity of the features used in this study and their occasional combination into prototypical bindings, drew from semantic memory, a function less affected by age than episodic memory (Nyberg et al., 1996). Future studies should investigate how much verbalisation or semantic memory can support performance on memory binding tasks that promote interactions between VWM and LTM.

In our study we presented participants with the encoding display once more before they entered the delay period. We did not predict that such a repetition would alter representations of the to-be-remembered items (Colzato et al., 2006; Treisman, 2006; see Logie et al., 2009). However, when Logie et al. (2009) probed participants with a recall procedure rather than with a yes/no recognition task, a substantial improvement in recalling colour-shape bindings was observed and small effects of learning on recall for colour-only, but not for shape-only, was also found. In the context of our task, we assessed memory reconstruction, a processes known to rely both on recognition (e.g., for object's parts) and on recollection (e.g., for the binding) (see Brockmole and Logie, 2013). It might be possible that such an effortful process may have resulted in more stable representations which survived the influence of longer delays. This influence appeared to have been strengthened by the availability of LTM which aided VWM performance (due to the familiar nature of features). This together with the availability of rehearsal mechanisms (the delay interval was unfilled) may have led to an improved performance after a longer delay. In terms of contextual demands, this task is less challenging than a pure recall task as it provides cues to retrieve the correct features (Parra et al., 2015; van Geldorp et al., 2015). However, it is more challenging than simple recognition during change detection tasks. Our prediction was that as active recognition/reconstruction processes would be more cognitive demanding than simple change detection, such a methodology would help investigate the actual extent of this age-insensitive function. Taken together these earlier findings and our own data they suggest that such insensitivity holds regardless of the memoranda and the retrieval function used to access it. Within the context of the present study we can claim that when it comes to VWM binding such mechanisms appear to remain available and fully functional as we grow older.

### Influences of education on VSTM binding across age

Education has proved one the greatest confounding factors in studies involving neuropsychological populations (Mungas et al., 2011), especially those suffering from dementia (Parra, 2014; Logie et al., 2015; Della Sala et al., 2016). Our group of older adults had significantly fewer years of education than our younger group, yet VWM binding functions were not differentially impaired in the former group (see Figure 3). When we controlled for the amount variance accounted for by Education, performance differences across groups decreased at the expense of changes in the younger but not in the older group (see Figure 4). From these observations it seems plausible to suggest that low education in old age does not impair those binding functions responsible for holding in VWM integrated features of everyday items whether in familiar or novel combinations. Parra et al. (2011) arrived to the same conclusions when in a *post-hoc* analysis they found that healthy subjects and patients with dementia due to AD from Colombia and the UK did not differ in their VWM binding abilities despite significant differences in the age and education of the samples collected across the two countries. In that earlier study the authors used arbitrary bindings of unfamiliar features, such as random polygons and non-primary colours. These results taken together suggest that is binding the function subserving these conjunctive memory mechanisms that is not affected by variables such as age and education and this seems to be true regardless of the nature of the to-be-bound information.

### Towards a culturally unbiased marker of cognitive ageing trajectories

The results presented here have important implications for the selection of culturally unbiased tests which can aid in the assessment of elderly people in countries with low socio-cultural backgrounds. A recent study has stressed on the cultural validity of the VSTM binding test (Della Sala et al., 2016). Here, we have shown that VSTM binding of features of everyday objects is preserved in older adults who have a low educational background. This creates new opportunities to incorporate these tools in the assessment of older adults at risk for dementia minimising the number of false positives and the need of using normative databases which have proved little informative to reveal the links between brain and behaviour across normal and abnormal ageing trajectories.

## Author contributions

MP, AG, and AM. designed the study. AG and AM. led the data collection. SH and MP. conducted the statistical analysis. SH, MP, AG, and AM. discussed the data. SH and AM. prepared the first draft. SH, MP, AG, and AM. generated the final draft.

### Conflict of interest statement

The authors declare that the research was conducted in the absence of any commercial or financial relationships that could be construed as a potential conflict of interest.
