# Supplementary material for: Feature Binding of Common Everyday Items Is Not Affected by Age
Source: Front Aging Neurosci. 2017 May 10;9:122. doi: 10.3389/fnagi.2017.00122 (PMC5423969; doi:10.3389/fnagi.2017.00122)
Supplement: Supplementary file 1 [file DataSheet1.DOCX]

***Supplementary Material***

**Feature binding of common everyday items is not affected by age and education: Towards a culturally unbiased marker of cognitive aging trajectories**

**Serge Hoefeijzers, Alfredis González Hernández, Angela Magnolia Rios, and Mario A Parra**

**Corresponding author:** Dr Mario A Parra, School of Social Sciences, Psychology Department, Heriot-Watt University, Edinburgh, EH14 4AS, [M.Parra_Rodriguez@hw.ac.uk](mailto:M.Parra_Rodriguez@hw.ac.uk)

| **Supplementary Table 1. Analysis of Congruency across item Category** | | | |
| --- | --- | --- | --- |
|  | **Living items**  Mean (SD) | **Man-made items**  Mean (SD) |  |
| **Young Adults (n=32)**  **Older adults (n=40)** | 94.92 (9.97)  93.75 (12.34) | 86.32 (10.21)  79.68 (17.20) |  |
| ***Note:*** We did not obtain congruency data from one older adult who felt a bit fatigue after the assessment. The data show that our indication of congruent stimuli is shared by both age groups. The effect of Category was significant (*F*(1,70) = 43.84, *p <* 0.001, η^2^_p_= 0.38; β= 1.00). Group had no significant effect (*F*(1,70) = 2.19, *p =* 0.144, η^2^_p_= 0.030, β=0.308). Importantly, the Group x Category Interaction was non-significant (*F*(1,70) = 2.55, *p =* 0.144, η^2^_p_= 0.035; β= 0.351). Hence, there is no evidence to think that the living and man-made category may influence memory performance across age. We therefore collapsed performance across this variable. | | | |

| **Supplementary Table 2.** Results of a 2 (age group) x 3 (test condition) ANOVA on the average accuracy scores (immediate and 30 sec delay scores collapsed), either controlled or not controlled for Education as covariate. | | | | |
| --- | --- | --- | --- | --- |
|  | **Without controlling for Education as covariate** | | **Controlling for Education as covariate** | |
|  | **Object-only, Colour-only, and Congruent Object-Colour binding conditions** | **Object-only, Colour-only, and Incongruent Object-Colour binding conditions** | **Object-only, Colour-only, and Congruent Object-Colour binding conditions** | **Object-only, Colour-only, and Incongruent Object-Colour binding conditions** |
| *Effect* |  |  |  |  |
| Group | ***F*(1,70)=49.822, p<0.001, η^2^_p_=0.416, β = 1.00** | ***F*(1,70)=54.944, p<0.001, η^2^_p_=0.440, β = 1.00** | ***F*(1,69)=11.327, p=0.001, η^2^_p_=0.141, β = 0.913** | ***F*(1,69)=13.042, p=0.001, η^2^_p_=0.159, β = 0.945** |
| Condition | ***F*(2,140)=23.123, p<0.001, η^2^_p_=0.248, β = 1.00** | ***F*(2,140)=6.182, p=0.003, η^2^_p_=0.081, β = 0.886** | ***F*(2,138)=7.694, p=0.001, η^2^_p_=0.100, β = 0.945** | *F*(2,138)=1.454, p=0.237, η^2^_p_=0.021, β = 0.307 |
| Group x Condition | ***F*(2,140) = 9.082, p<0.001, η^2^_p_=0.115, β = 0.973** | *F*(2,140)=0.517, p=0.598, η^2^_p_=0.007, β = 0.134 | ***F*(2,138)=3.629, p=0.029, η^2^_p_=0.050, β = 0.662** | *F*(,)= 0.340, p=0.712, η^2^_p_=0.005, β = 0.104 |
|  | | | | |

| **Supplementary Table 3.** Results of a 2 (age group) x 2 (binding congruency - congruent vs. incongruent Object-Colour binding) ANOVA collapsing across delays and when Education was either controlled or uncontrolled. | | |
| --- | --- | --- |
|  | **Without controlling for Education as covariant** | **Controlling for Education as covariant** |
| *Effect* |  |  |
| Age | ***F*(1,70)=21.905, p<0.001, η^2^_p_=0.238, β = 0.996** | *F*(1,69)=2.808, p=0.098, η^2^_p_=0.039, β = 0.379 |
| Binding congruency | ***F*(1,70)=11.111, p=0.001, η^2^_p_=0.137, β = 0.908** | ***F*(1,69)=9.347, p=0.003, η^2^_p_=0.119, β = 0.854** |
| Age x Binding congruency | ***F*(1,70)=14.307, p<0.001, η^2^_p_=0.170, β = 0.962** | *F*(1,69)=3.258, p=0.075, η^2^_p_=0.045, β = 0.429 |
|  | | |

**Supplementary Figure 1: Effect of test-item (living vs. non-living) on memory binding for congruent and incongruent test material.**

Recognition accuracy is not significantly different between living and non-living items (no main effect of test item: *F*(1,69) = 3.465, p = 0.067, η^2^_p_ = .048, β=0.451). In addition, the effect of test item (i.e. living vs non-living items) on recognition accuracy did not differ between groups (no test item * group interaction: *F*(1,69) = 0.925 p = 0.339, η^2^_p_ = .013, β=0.158), test condition (no test item * test condition interaction: *F*(1,69) = 0.178, p = 0.675, η^2^_p_ = .003; β =0.070), or delay (No test item * delay interaction: *F*(1,69) = 0.059, p = 0.808, η^2^_p_ = .001, β=0.057).

**Note:** Data has been controlled for education (i.e. variable “education” was inserted as covariate) as level of education differs significantly between groups (*t*(48.52) = 6.80, p < 0.001, *r* = .70, β=1.00) and education has a significant effect on recognition accuracy (*F*(1,69) = 10.864, p = 0.002, η^2^_p_ = .136, β=0.901).
